# CD4^+^CD25^high^CD127^low/−^FoxP_3_^+^ Regulatory T Cell Subpopulations in the Bone Marrow and Peripheral Blood of Children with ALL: Brief Report

**DOI:** 10.1155/2018/1292404

**Published:** 2018-05-29

**Authors:** M. Niedźwiecki, O. Budziło, M. Zieliński, E. Adamkiewicz-Drożyńska, L. Maciejka-Kembłowska, T. Szczepański, P. Trzonkowski

**Affiliations:** ^1^Department of Pediatrics, Hematology and Oncology, Medical University of Gdansk, Gdańsk, Poland; ^2^Clinical Immunology and Transplantology Unit at the Department of Immunology, Medical University of Gdansk, Gdańsk, Poland; ^3^Department of Pediatric, Hematology and Oncology, Zabrze Medical University of Silesia, Katowice, Poland

## Abstract

CD4^+^CD25^high^CD127^low/−^FoxP_3_^+^ regulatory T cells (Tregs) are currently under extensive investigation in childhood acute lymphoblastic leukemia (ALL) and in other human cancers. Usually, Treg cells maintain the immune cell homeostasis. This small subset of T cells has been, in fact, considered to be involved in the pathogenesis of autoimmune diseases and progression of acute and chronic leukemias. However, whether Treg dysregulation in CLL and ALL plays a key role or it rather represents a simple epiphenomenon is still a matter of debate. Treg cells have been proposed as a prognostic indicator of the clinical course of the disease and might also be used for targeted immune therapy. Our study revealed statistically higher percentage of Treg cells in the bone marrow than in peripheral blood in the group of 42 children with acute lymphoblastic leukemia. By analyzing Treg subpopulations, it was shown that only memory Tregs in contact with leukemic antigens showed statistically significant differences. We noticed a low negative correlation between Treg cells in the bone marrow and the percentage of blasts (*R* = −0.36) as well as a moderate correlation between Treg cells in the bone marrow and Hb level (*R* = +0.41) in peripheral blood before therapy. The number of peripheral blood blasts on day 8th correlates negatively (*R* = −0.36) with Tregs. Furthermore, statistical analysis revealed low negative correlation between the number of Tregs in the bone marrow and the minimal residual disease measured on day 15th, the percentage of blasts in the bone marrow and leukocytosis after 15 days of chemotherapy. These results indicate the influence of Tregs on the final therapeutic effect.

## 1. Introduction

Acute lymphoblastic leukemia (ALL), the most common childhood cancer, is a heterogeneous disease that occurs due to the malignant clonal proliferation of lymphoid progenitors [1]. The clinical symptoms of the disease and the ultimate therapeutic effect depend on the biological characteristics of the tumor cell. Another very important factor in curing cancer is an efficient immune system. Despite intensive research on the effects of various elements of the immune system on the cancer development, there is still little knowledge about it.

Normal cells in the environment of cancer cells are currently under intensive investigation. Residual nonmalignant T cells and B cells are in permanent cell-to-cell contact with lymphoblasts and are involved in active immune responses [2].

Regulatory lymphocytes constitute a very interesting subpopulation of cells of the human immune system. A growing interest in their biological properties has occurred recently and clinicians have wondered whether they can be also used in the battle against cancer [3, 4].

Recent papers have demonstrated elevated number of Tregs in lung, breast, pancreatic, ovarian, melanoma, digestive system cancers, CLL, T cell ALL, and B cell NHL [1, 5, 6]. This concerns both peripheral blood and cancer tissue, where a neoplastic proliferation is accompanied by higher than the usual number of regulatory lymphocytes. In some subtypes of cancer, the differences in the percentage may affect the response to chemotherapy and thus the prognosis of a disease.

It was demonstrated previously that elevated percentages and increased suppressor properties of Treg cells are observed even after achieving a remission and after completing the treatment of AML [6]. This might indicate that Tregs are resistant to chemotherapy and could facilitate a relapse of AML.

Previous research showed that the number of Treg cells may be either elevated or reduced in Hodgkin disease and mature B cell lymphoma. Similarly, a prognosis may be either favorable or adverse [7]. It is known for example that the percentage of Treg cells is higher among patients suffering from CML than among healthy volunteers [8]. This level correlates with an advancement of the disease, the percentage of B cells in peripheral blood, and the level of LDH. Some papers even state that Treg cells may control a neoplasmatic growth [9]. The next interesting issue is a connection between Treg cells and ALL among patients in the developmental age. This group of leukemias are characterized by a separate biology, clinical picture, and first of all—different prognosis.

In our study, we investigated a population of CD4^+^CD25^high^CD127^low/–^FoxP_3_^+^ regulatory T cells in the bone marrow and peripheral blood of children with acute lymphoblastic leukemia treated in the Department of Pediatrics, Hematology and Oncology, Medical University of Gdansk in 2011–2016.

Due to the small number of publications concerning the influence of Tregs on the prognosis in acute childhood leukemias and investigating the percentage of these cells in the bone marrow and peripheral blood of ALL children, a following research was undertaken to understand these relationships better.

Of particular interest to us was the influence of a higher percentage of Tregs in the peripheral blood/bone marrow of patients with acute leukemia on the early and late therapeutic effect, which was reported in the literature [10, 11].

In addition, it was decided to perform an initial assessment of the relationship between biological characteristics of leukemia and Tregs. By assessing the correlation between the number of Tregs and such parameters as hemoglobin, platelets, leukocytosis, or the percentage of blasts in the peripheral blood and bone marrow at the moment of diagnosis, it was decided to verify the preliminary hypothesis which assumes the connections between Tregs and the stage of the cancer process and prognosis. The elevated percentage of Treg cells in the bone marrow observed earlier by some authors in comparison to peripheral blood requires verification due to the use of a narrow panel of antibodies to assess the population of cells of interest. To effectively and reliably count Treg cells in the analyzed material, antibodies were used to identify cells with the CD4^+^CD25^high^CD127^low/−^FoxP_3_ phenotype, which identifies the T-line regulatory cells in the most accurate way [4].

In case the relationships described earlier in the literature were confirmed, it would be quite advisable to search for therapeutic methods interfering with the immune system through manipulations on Treg cells [9].

An interesting question was also whether the increased percentage of Tregs in the peripheral blood and/or bone marrow is also observed in children with cancers other than leukemia [12].

In summary, Tregs are a potential target of immunotherapy but this hypothesis requires further, intensive investigation of the properties of relationships between regulatory and cancer cells. This could contribute to the improvement of a prognosis with simultaneous reduction of toxic chemotherapy [9].

## 2. Methods

### 2.1. Patients and Treatment

The bone marrow and peripheral blood were obtained at diagnosis from 42 patients with acute lymphoblastic leukemia treated according to the BFM SG Protocol ALL-IC BFM 2002 (*n* = 1) and Protocol ALL IC-BFM 2009 (*n* = 41).

All clinical data concerning patients are summarized in Table 1.

This study was approved by the Medical University of Gdansk Ethical Board, and informed consent was obtained from patients and/or their legal guardians.

A response to the steroid therapy was checked in the peripheral blood on day 8, a remission in the bone marrow was checked by the flow cytometry on day 15 and 33. Patients were divided to SR, IR, and HR risk groups according to the protocol rules.

### 2.2. Control Group

In the control group, 10 bone marrow samples (2 ml) and 46 peripheral blood samples (5 ml) were tested. For ethical reasons, the bone marrow was obtained only from the children requiring a diagnostic bone marrow biopsy to exclude bone marrow involvement by a cancerous disease or to exclude leukemia. In the control group, the following diagnoses were noted: Wilms tumor (*n* = 9), neuroblastoma (*n* = 9), RMS (*n* = 2), Hodgkin disease (*n* = 11), CNS tumor (*n* = 5), anemia (*n* = 3), and lymphadenopathy (*n* = 7).

The analysis involved 24 girls and 22 boys from 1 to 16 years of age.

### 2.3. Regulatory T Cell Immunophenotyping by Multicolor Flow Cytometry

Only freshly obtained samples were processed up to 24 hours from collection. Briefly, lymphocytes were isolated using density gradient media Lymphoprep (STEMCELL Technologies, Canada) and EDTA bone marrow or peripheral blood samples. Lymphocytes were then stained with the use of CD127 FITC (clone HL-7R-M21), CD25 PE (clone 2A3), CD4 PerCP (clone SK3), CD3 V450 (clone UCHT1), CD45RA PEcy7 (clone L48), and CD62L Alexa Fluor750 (clone Dreg-56). All of the antibodies were obtained from BD Bioscience, USA, except for CD62L from Life Technologies, USA. Permeabilization was done with the use of Foxp3 Staining Buffer Set Kit (eBioscience, USA), while for intracellular staining, FoxP_3_ APC was used (clone PCH 101, eBioscience, USA). The readout was done with BD FACSCANTO II (BD Bioscience, USA) and 100.000 of cells were acquired.

**Figure 1:**
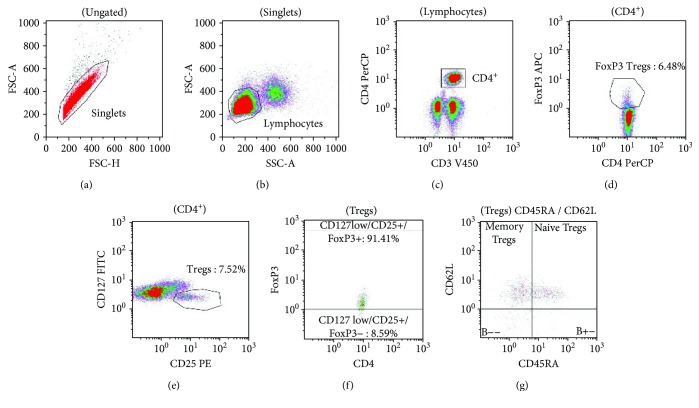


A representative example of Treg subpopulations gating is given in Figure 1.

### 2.4. Gating Strategy

First, singlets were identified according to FSC area to height signal distribution (A). Then lymphocytes (B) and CD3+/CD4+ T lymphocytes were gated (C). Next, regulatory T cells were identified as CD4+/FoxP_3_ double-positive T cells (D), as well as CD127low/CD25+ T cells (E). To get the best overlay between CD127low/CD25+ and FoxP_3_, Treg gate was put to get minimum 90% of cells in that were FoxP_3_ positive (F). Then another gate was plotted to identify naïve Tregs as CD45RA+/CD62L+ and memory Tregs as CD45RA–/CD62+ T lymphocytes (G).

### 2.5. Statistical Analysis

Clinical data, laboratory findings, and family history of the disease were collected in the medical database constructed in Microsoft Excel software for Windows 10 (Microsoft). Data were analyzed using Statistica software version 7.1 for Windows (StatSoft Inc. 2005). Shapiro-Wilk test was used to estimate either normal or abnormal spread of analyzed variables. Depending on the spread of variable, nonparametric Mann–Whitney *U* test, ANOVA Kruskal-Wallis test, Wilcoxon test, ANOVA Friedman test, and parametric Student's *t*-test were used. Chi-square test and estimation of the correlation (R Spearman, Pearson) were used for statistical analysis of some variables. Significance level was *p* < 0.05.

## 3. Results

In our study, for the first time, the percentage of individual subpopulations of regulatory T cells (Tregs) among CD3+CD4+ lymphocytes in the bone marrow and peripheral blood of children suffering from acute lymphoblastic leukemia were determined (Table 2).

### 3.1. Regulatory T Cell in the Bone Marrow and Peripheral Blood at Diagnosis of Childhood ALL

#### 3.1.1. Comparison of Treg Number in the Bone Marrow and Peripheral Blood in the Group of Children with Acute Lymphoblastic Leukemia (Figure 2)

**Figure 2:**
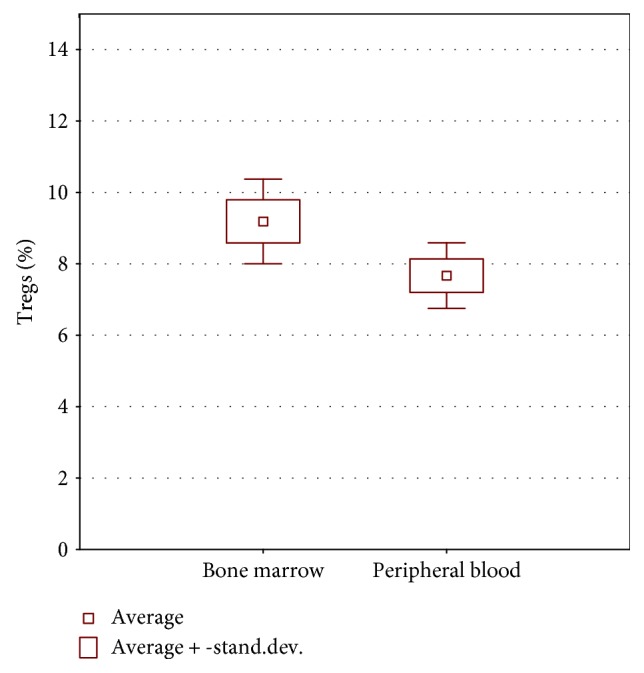
Bone marrow and peripheral blood percentage of Tregs in CD4+ population of cells among children with ALL.

Percentages of regulatory T cells were significantly higher in the bone marrow (9.59+\–3.58) as compared to the peripheral blood (7.81+\–2.73) (*p* = 0.002) (Figure 2). This was not demonstrated in the group of children with diagnosis different than acute leukemia (solid tumors, healthy children) (*p* = 0.83), but the size of this group was quite small. The observations above might be caused by the natural tendency of Treg cells to accumulate in the bone marrow in a higher percentage than in the peripheral blood.

#### 3.1.2. Comparison of Treg Level in the Bone Marrow/Peripheral Blood among Children with Acute Lymphoblastic Leukemia versus Solid Tumors/Healthy Children

**Figure 3:**
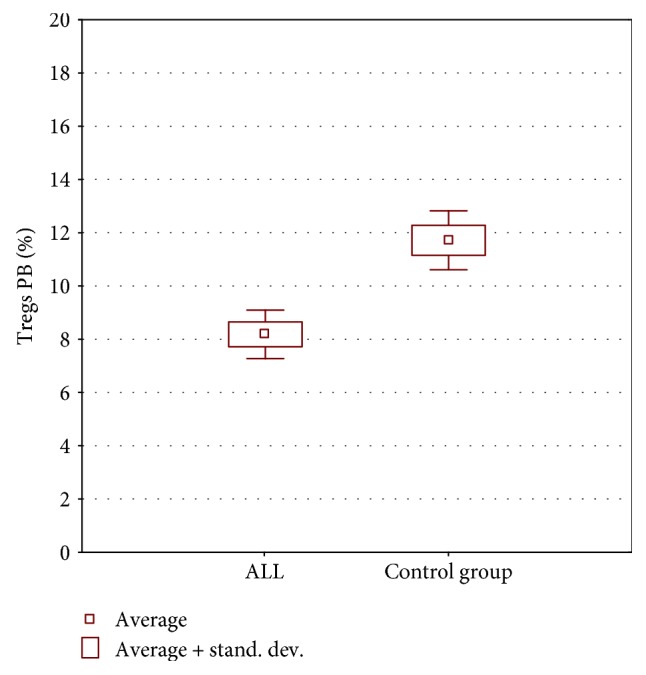
Treg level in peripheral blood among children with ALL versus control group.

When the percentage of Tregs in the bone marrow was measured, the analysis showed no statistically relevant differences between children suffering from ALL and those diagnosed with solid tumor/anemias/lymphadenopathy or even healthy ones. But when the peripheral blood was taken under investigation, there was statistically higher percentage of Tregs among children in the control group in comparison to pediatric patients diagnosed with ALL (Figure 3). Due to the small size of the control group and recognized diseases that may affect the percentage of Treg cells in the bone marrow and peripheral blood, the obtained results are not very reliable. However, the statistically significant differences obtained indicate clearly the need to repeat the analysis among patients with solid tumors.

#### 3.1.3. Statistical Analysis of Selected Treg Subpopulation in Analyzed Population of ALL Children

**Figure 4:**
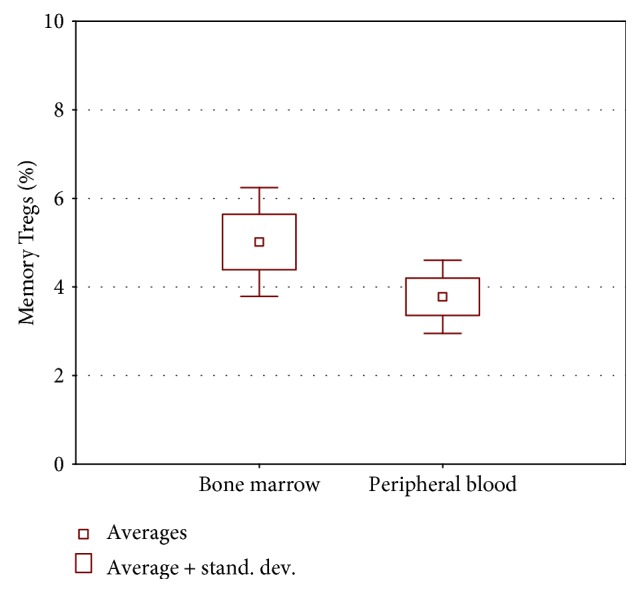
Statistical analysis of selected Treg subpopulations in analyzed population of ALL children.

Memory Treg proportion in the bone marrow of children with ALL was statistically higher than the percentage in peripheral blood (*p* = 0.006) (Figure 4), while percentages of natural naïve Tregs (*p* = 0.63) and induced Tregs (*p* = 0.26) did not differ between blood and bone marrow.

#### 3.1.4. Risk Factors and Biological Characteristic of Cancer Cell versus Tregs Subpopulation in Group of 42 Children with ALL

To identify prognostically relevant parameters, we analyzed correlation between Treg subpopulations and ALL well-known risk factors and a response to treatment. The risk factors and disease parameters analyzed in our study were gender, age, leukocytosis, and blastosis in the peripheral blood, bone marrow blast count at diagnosis, CNS status (M1; M2; M3 according to Protocol ALLIC 2009), and initial qualification to the risk group (SR, IR, and HR).

The response parameters analyzed in our study were blasts' sensitivity for prednizon (number of blasts on the 8th day of therapy), the percentage of blasts in the bone marrow on day 15 and 33 (<5% (M1 status) or 5% to 20% (M2) or >20% (M3)), and white blood cell count in peripheral blood on day 15 and 33.

**Figure 5:**
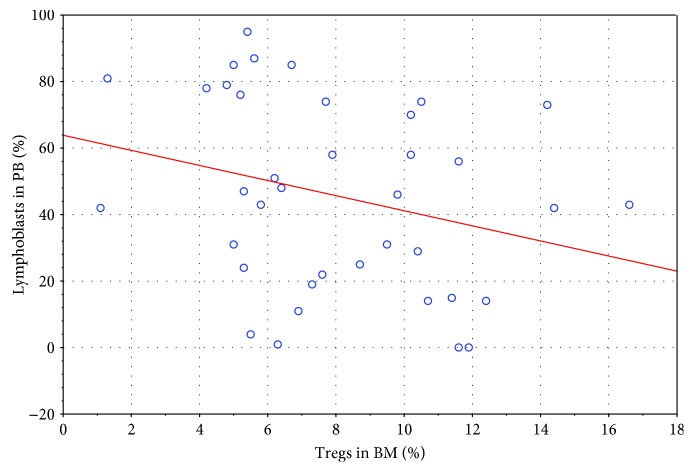
Correlation between the percentage of Tregs in BM and percentage of blasts in PB at the moment of diagnosis (*R* = –0.36).

**Figure 6:**
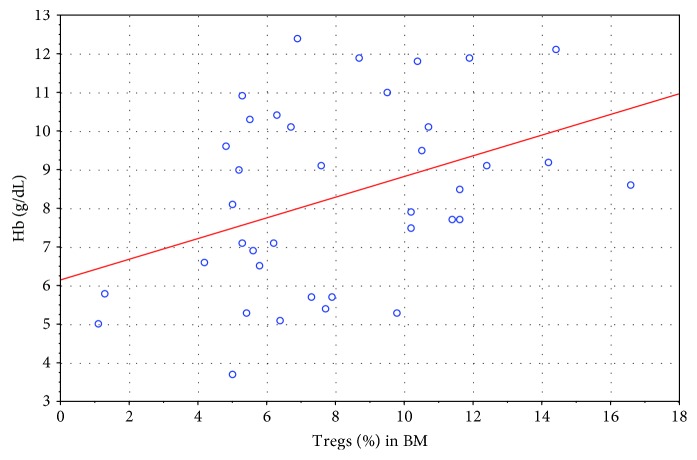
Correlation between the percentage of Tregs in BM at the time of diagnosis with the hemoglobin level (*R* = +0.41).

**Figure 7:**
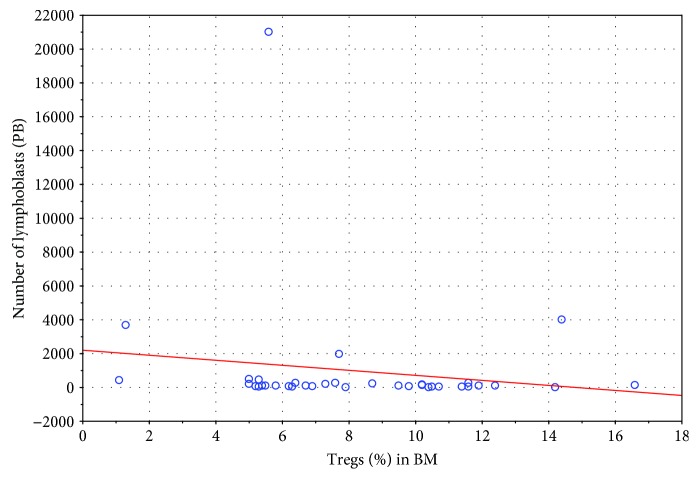
Correlation between blasts level on the 8th day of steroid therapy in PB with the percentage of Tregs in BM at the moment of diagnosis (*R* = –0.36).

Statistical analysis revealed a few interesting observations. A low negative correlation was noticed between Treg cells in the bone marrow and the percentage of blasts in peripheral blood (Figure 5; *R* = −0.36) as well as a moderate correlation between the first one and Hb level in PB before therapy (Figure 6; *R* = +0.41). At the same time, peripheral blood blasts level on day 8 correlates negatively low with the number of Tregs in BM at the moment of diagnosis (Figure 7; *R* = −0.36).

#### 3.1.5. Answer for the Chemotherapy according to Protocol BFM ALLIC 2009

Statistical analysis revealed a low negative correlation between the level of Tregs in the BM and the minimal residual disease measured on day 15 (MRD 15; *R* = –0.24), as well as the percentage of blasts in the bone marrow (*R* = –0.24) and leukocytosis (*R* = –0.2) after 15 days of chemotherapy.

## 4. Discussion

ALL is one of the most common childhood cancer with favorable prognosis. Less than 20% of children with acute lymphoblastic leukemia have unfavourable prognosis and suffer from a relapse, resistant ALL, or serious complication of the chemotherapy [13]. For children with recurrent or refractory leukemia, new therapeutic options based on molecular biology and immunological therapy must be found to avoid serious and mortal complications caused by a high-dose chemo- and radiotherapy [14].

Treg cells play a key role in human immunological reaction towards the neoplasmatic cells in the organism. An increased number of Treg cells was noticed in many solid tumors, for example, breast, colon, and lung tumors [7]. In most of the hematological malignances, D'Arena et al. in 2011 noticed that Treg cells numbers were elevated in peripheral blood and correlated with the stage of a disease and the prognosis [15].

Generally in human cancers, higher percentages of Tregs predict worse immunological reaction to the viral infection and cancer antigens. However, the role of Treg cells in the pathogenesis of ALL and AML is still unclear [6].

Tregs are defined on the basis of combined expression of CD4, CD25, FoxP_3,_ low expression of the CD127, and CD4^+^CD25^high^CD127^low/–^FoxP_3_^+^ regulatory T cell phenotype is the most appropriate one. This sensitive and reliable phenotype was used to determine the percentage of these cells in the bone marrow and/or peripheral blood of children with ALL.

So far, no data has been reported on Treg cell number in the bone marrow among children with acute leukemias. Similarly, very limited evidence is reported about Treg cells in children leukemias and the influence of their number on the prognosis and their correlation with already known risk factors. Lustfeld et al. suggest that elevated proportions of CD4+ T cells among residual bone marrow T cells in ALL is associated with favorable early responses [10]. Several other authors have come to similar conclusions, among others Szczepanski et al. [6] in AML or Idris et al. [1] in ALL.

Our analyses confirm these observations and indirectly indicate a correlation between the percentage of Tregs and prognosis in pediatric ALL. The correlations between all risk factors and hematological parameters of ALL patients and peripheral blood and bone marrow Treg number were analyzed. Probably due to the small size of the group, it was possible to detect only the low negative correlation (*R* = −0.36) between bone marrow Tregs (%) and the number of blasts in peripheral blood after 8 days of steroid therapy. As it is known, blastosis after eight days of steroid therapy is one of the most important prognostic factors in acute lymphoblastic leukemia and often correlates with the response to chemotherapy after 15 and 33 days of intensive treatment.

Thus, the correlation between blasts' sensitivity to steroids and the percentage of Tregs in the bone marrow of children with ALL leukemia is most likely the evidence of the prognostic significance of Tregs for the prognosis of cure. However, if the thesis above would be too daring, then undoubtedly Tregs have at least a significant influence on the response to administered steroids.

It is therefore reasonable to assume that by interfering with immune regulatory system, there might be a possibility to influence the effectiveness of the therapy used, which would consequently lead to the reduction of the dose of therapy without affecting the final therapeutic answer [16]. The lack of correlation between regulatory cells and MRD on day 15, as well as the rate of blasts on 15 and 33 days is primarily associated with the abolition of Treg level as a response to treatment with the appropriately selected chemotherapy. Therefore, the most important prognostic factor in acute childhood leukemia is an appropriately selected risk group for intensive chemotherapy.

At the time of diagnosis, Treg level in the bone marrow also showed a low negative correlation (*R* = −0.36) with tumor cell levels in peripheral blood. The latter is an important prognostic factor in ALL. The percentage of blasts found in blood at diagnosis is indirectly indicative when it comes to severity and malignancy of the cancer. Vigorè et al. [17] discovered the dependence of the percentage of Tregs during the progression of cancer on the presence of metastatic changes in various types of solid tumors. Statistically, a higher percentage of regulatory cells in the peripheral blood was detected among patients with advanced cancer than those with no metastatic changes. This may indicate the inhibitory effect of Tregs on the effector anticancer arm of the immune system.

Initially, the most important aim of our study was to confirm the observation of an increased percentage of Treg cells in the bone marrow and/or peripheral blood of patients with acute leukemia made by other researchers [1, 6, 11, 13, 18].

The tumor microenvironment, especially suppression of tumor-associated antigen-reactive lymphocytes, is an important factor in the development and progression of cancer [19]. Recent evidence suggests that the cellular composition of the tumor microenvironment, particularly the quantity of the tumor-infiltrating Tregs, can significantly modify the clinical outcome in hematologic malignancies, particularly in some subtypes of lymphomas. Tregs are one of the most interesting populations of immunologically competent cells engaged in fighting cancers [14, 20, 21]. So far, more evidence indicates that regulatory lymphocytes migrate to some particular sites in need of immune regulation [22].

Our study revealed statistically higher percentage of Treg cells in the bone marrow than in peripheral blood in the group of 42 children with acute lymphoblastic leukemia. This fact is a very interesting discovery in the context of described infiltration of regulatory lymphocytes into the neoplasmatic tissue in some types of tumors and hematological malignancies. In case of leukemia, such tissue is bone marrow. A similar relationship in the group of patients diagnosed with other condition than acute lymphoblastic leukemia has not been discovered. Hence, there is our interest in these cells in terms of risk factors and biological features of leukemia cells.

It is not known for sure whether the elevated Tregs in the bone marrow is the response to hematological malignancies or the cause of a developing cancer. It is also unclear whether the percentage of regulatory cells correlates with the recognized prognostic factors in acute lymphoblastic leukemia in children. It was only noted that one study on the murine models showed a correlation between the progression of cancer and the migration of regulatory cells into the tumor tissue [22, 23]. On the other hand, in certain cancer, such as colorectal carcinoma, Tregs suppress bacteria-driven inflammation which promotes carcinogenesis [24, 25]. In this situation, Treg level is an important risk factor. According to some researchers, the tumor-infiltrating, immune-competent regulatory lymphocytes have a great impact on the prognosis. Thus, they might be a very interesting therapeutic option and they need to be determined for each type of cancer separately [15, 16]. Undoubtedly, further research must evaluate this issue.

Another cause of elevated number of lymphatic regulatory cells in the bone marrow of children with acute leukemia may be their natural tendency to accumulate in this tissue [26]. According to some authors, the higher percentage of Treg cells in the bone marrow compared to peripheral blood is natural and is associated with factor CXCL12 [26]. Bone marrow strongly expresses functional stromal-derived factor (CXCL12), the ligand for CXCR4. CXCR4/CXCL12 signals are crucial for Treg homeostasis in the bone marrow and are responsible for the observed effect both in the sick and healthy bone marrow [26].

The question remains whether the increased percentage of Treg cells in the bone marrow compared to peripheral blood in children with ALL results from the physiological tendency of Tregs to accumulate in the bone marrow or from the direct contact of immunocompetent cells with blasts.

The study led us to accept the thesis that a higher proportion of Tregs in the bone marrow of children with acute leukemia is due to the interaction of leukemic cells with Tregs. It is less likely that the accumulation of Treg cells in the bone marrow in a such high percentage is a physiological phenomenon.

Interestingly, it seems that there are no statistically significant differences in the proportion of Tregs between BM and PB in patients who suffer from tumors other than ALL [21, 26, 27].

Detailed analysis of Treg subsets showed very interesting features associated with ALL. The bone marrow of children with ALL was infiltrated by a higher percentage of memory Tregs than the peripheral blood. There was no difference in the number of naive Tregs in both peripheral blood and the bone marrow.

Memory Tregs arise after contact with their own antigen [28]. Undoubtedly leukemia cells express on their surface and cytoplasm antigens identical to the antigens found in the healthy cells. Therefore, in our opinion, the population which is observed is natural regulatory lymphocytes that have been formed after the contact of naive regulatory cells with antigens present on leukemic and normal cells.

The site of this transformation is probably the bone marrow as the percentage of memory Tregs was the highest there. Alternatively, memory Tregs are formed at the periphery and traffic to the sites with high expression of their cognate antigen, such as the leukemic bone marrow.

Most likely, Tregs accumulate there to exert the suppressive effect on the proliferating leukemic blasts. Unfortunately, the increasing percentage of Tregs suppresses also the immune system, which tries to fight a developing tumor. This is probably the reason why the increased percentage of Tregs is seen at the periphery at the very advanced stages of ALL (not analyzed here), while it might be favorable in leukemic bone marrow at early stage of the disease.

Hence, some manipulation on Tregs might be considered as a part of the treatment of hematological malignancies [19, 29, 30].

## 5. Conclusion

Regulatory T lymphocytes are group of cells that might play important role in the development of cancerous diseases including acute leukemia in children. Their elevated bone marrow and peripheral blood rate among children diagnosed with ALL might be linked to the development of the disease. Manipulations involving Tregs might represent an interesting therapeutic option and may be used to enhance the effect of antitumor chemotherapy. Larger studies are now warranted to validate these findings and determine their clinical implications.

## Figures and Tables

**Table 1:** Patient characteristics (*n* = 42).

Age	1–5 years = 26	6–11 years = 10	12–18 years = 6
Gender	Male = 21	Female = 21	
Immunophenotype	B cell = 40	T cell = 2	
Protocol of therapy	ALL IC BFM 2002: 1	ALL IC BFM 2009: 41	
Risk group	SR: 5	IR: 25	HR: 12
CNS involvement	Positive: 2	Negative: 40	
EFS	Relapse: 2	Death: 2 (after relapse: 0)	Live in first remission: 38
Steroid sensitivity^∗^	Good: 35	Poor: 6	
BM on day 15^∗^	M1: 28	M2: 9	M3: 4
BM on day 33^∗^	M1: 38	M2: 1	M3: 2

^∗^One child died before the 8th day of the treatment, so we were not able to assess the sensitivity to steroids and the response to treatment on the 15th and 33rd days of chemotherapy.

**Table 2:** Distribution of tested parameters in the bone marrow and peripheral blood in children at diagnosis of ALL.

Tested parameters	Average (%)	Min-max (%)	(Event count) min-max	SD
Treg BM	9.59	2.23–19.03	3528–9913	3.58
Treg PB	7.81	3.33–13.36	2183–6942	2.73
Natural Treg BM	5.39	1.00–16.40	1574–7391	3.80
Natural Treg PB	3.85	0.70–12.00	867–5855	2.38
Natural naive Treg BM	3.86	0.10–8.20	569–4003	1.98
Natural naive Treg PB	3.80	0.90–9.50	1161–5814	2.14

## Data Availability

The statistical analysis (as Excel and Statistica files) used to support the findings of this study are available from the corresponding author upon request.

## Abbreviations

Tregs: Regulatory T cells
BM: Bone marrow
PB: Peripheral blood
Hb: Hemoglobin
ALL: Acute lymphoblastic leukemia
CLL: Chronic lymphoid leukemia
NHL: Non-Hodgkin lymphoma
AML: Acute myeloid leukemia
RMS: Rhabdomyosarcoma
LDH: Lactate dehydrogenase
BFM SG: Berlin-Frankfurt-Munster study group
SR: Standard risk (group)
IR: Intermediate risk (group)
HR: High risk (group)
EFS: Event-free survival
MRD: Minimal residual disease
CNS: Central nervous system
CD: Cluster differentiation antigen
FoxP3: Forkhead box P3
CXCL12: Stromal cell-derived factor 1
CXCL4: Platelet factor 4.
